# Modelling district heating demand: A synthetic dataset for two residential neighbourhoods

**DOI:** 10.1016/j.dib.2025.111294

**Published:** 2025-01-13

**Authors:** Katia Ritosa, Ina De Jaeger, Dirk Saelens, Staf Roels

**Affiliations:** a KU Leuven, Department of Civil Engineering, Building Physics and Sustainable Design, Kasteelpark Arenberg 40, BE-3001 Leuven, Belgium; b EnergyVille, Thor Park 8310, BE-3600 Genk, Belgium

**Keywords:** Artificial dataset, Simulated neighbourhood, Energy demand, Thermal behaviour

## Abstract

The extensive artificial datasets developed in this study capture the energy demands of two districts and, with reasonable constraints, emulate monitoring campaigns typically conducted on-site in inhabited houses. Generated datasets are the following, one 1) representing low-performing building stock from before the deployment of the Energy Performance of Buildings Directive (EPBD) (2006), and the other 2) reflecting high-performing stock constructed after 2006. The buildings in the datasets were simulated representing single-family homes, typical of neighbourhoods in Flanders, Belgium. The datasets were generated using Dymola and the IDEAS package integrated with TEASER. Each simulated house differs in geometry, size, envelope characteristics, occupancy patterns, and installed gas heating systems. Envelope characteristics for older houses were derived from Energy Performance Certificate (EPC) data, and categorized into four construction periods, while newer houses were based on Energy Performance of Buildings (EPB) reports, both evaluated in Flanders. The datasets feature only heavy-weight houses in terraced, semi-detached or detached configurations, modelled as either single- or two-zone buildings, depending on their number of storeys. The simulations incorporate a natural infiltration model and a stochastic occupant behaviour model that sets the temperature requirements and accounts for heat gains from occupants and appliances. Due to the computational effort of large-scale simulations, heating system performance was postprocessed using a data-driven approach assuming gas-fired heating systems. Six heating system configurations were allocated, including condensing and non-condensing boilers, each combined with one of three domestic hot water (DHW) setups: no integrated DHW, direct DHW, and DHW with a storage tank. For all system designs, production efficiency varied with the load ratio. Urban-scale simulations were carried out at 10-minute frequency using weather data for Heverlee, Belgium, from the year 2016.

The primary objective of this dataset was to support the development of statistical tools for characterizing the heat transfer coefficient of building envelopes. However, the generated artificial datasets offer a wide range of typically hard-to-measure inputs, making them ideal for evaluating the impact of simulated components on the overall energy balance. While the initial focus was on the behaviour of individual buildings, these datasets are also valuable for urban-scale analyses, particularly in energy planning contexts.

Specifications Table

**Table:** 

Subject	Energy
Specific subject area	Simulation of heating demand for residential building neighbourhoods
Type of data	Table (.csv format)
Data collection	The data was created carrying out building energy simulations in the modelling tool Dymola with the help of the IDEAS package embedded in the TEASER framework.
Data source location	Institution: KU Leuven, Building Physics and Sustainable Design Section at the Civil Engineering Department Country: Belgium
Data accessibility	Repository name: Zenodo Data identification number: 10.5281/zenodo.14191440 Direct URL to data: 10.5281/zenodo.14191440
Related research article	K. Ritosa, D. Saelens, and S. Roels, “A large scale artificial dataset to evaluate the impact of on-board monitoring set-ups on the Heat Transfer Coefficient assessment,” Energy Build., vol. 289, p. 113061, Jun. 2023, 10.1016/j.enbuild.2023.113061

## 1 Value of the Data

• The presented two artificial neighbourhoods reflect realistic variations in building properties and behaviour present in inhabited homes and these comprehensive datasets mimic monitoring campaigns usually collected on-site. The simulation inputs reflect the building characteristics and occupancy preferences in Belgian (Flemish) homes. Starting from the idea of assessing the existing building stock, i.e. low-performing buildings constructed before introducing rigorous standards in 2006, the simulated framework also extends newly built and well-performing buildings as the overall thermal behaviour, including the heat demand and the importance of internal gains and losses. Providing a large spectre of demand profiles, the simulated buildings can be investigated as individuals, clustered in smaller units (i.e. streets), or the trends can be compared between neighbourhoods.
• By allocating the system characteristics in postprocessing, the benefits are twofold. The original simulation outputs provide the ideal space heating and domestic hot water demand and allow users alternative types of data postprocessing. In case the heating system postprocessing is taken into account, the provided output includes an assumed emission, distribution, control, and storage efficiency, as well as a load-dependent production efficiency for 6 gas-fired heating system typologies. When postprocessing is considered, the least detailed heating input measurement resembles raw data collected after a smart gas meter.
• Three types of datasets are provided: 1) datasets containing general information on the building characteristics and occupancy (e.g. construction year, house type, number of occupants, …), 2) the weather file, and 3) individual datasets with the energy simulation outputs for every building. The outputs of the simulation comprise amongst others the temperature measurements, net heating demand, and internal gains for each zone, while the overall heating demand is provided on the building level on different measurement levels of detail and includes the domestic hot water demand. In total, datasets for 1086 low-performing (<2006) and 1021 well-performing (>2006) houses are made available.
• The created artificial data can mimic various monitoring setups reflecting a variable level of detail, ranging from detailed to limited datasets. Thus, they enable the comparison of multiple scenarios and setups in the same boundary conditions, including both occupancy and weather, which is not achievable in real life. The created datasets present a useful resource because they can resemble perfect yet realistic monitoring campaigns. To produce similar high-quality datasets using real-life measurements would be unfeasible in practice and would require substantial effort in tailoring and executing measurement campaigns.
• The generated datasets can serve as the foundation for future research involving building performance evaluation, urban building energy models, urban planning, energy management, integration of renewables and smart grids, and the development of various data-driven and statistical models.

## 2 Background

The primary motivation behind creating these datasets was the lack and complexity in gathering large-scale monitoring campaigns that capture the heating demand and thermal behaviour of single-family residential buildings. Therefore, comprehensive simulated datasets were chosen as a suitable alternative. These datasets served as input for developing data-driven methods for characterizing building thermal performance indicators, such as the heat transfer coefficient (HTC) of the building envelope.

To develop statistical tools and a methodological framework in [1], artificial data served as a reliable foundation since sensor inaccuracy was not reflected in the measurements, and the systematical selection of any simulation variables as inputs to the statistical tools was enabled (which is never achieved in real life). Such an approach also allowed the selection of an ‘ideal’ monitoring set-up which considered all components of the common heat balance equation and estimated their effect on the examined indicators (HTC). Furthermore, by adopting artificial houses the presumed estimation target was unanimously defined and it enabled the estimation of the statistical accuracy, along with the robustness and reliability of the approach on a large and diverse sample. The same districts were used also in [2,3].

## 3 Data Description

In this article, two sets of data reflecting the thermal behaviour and energy requirements for one low-performing and one well-performing district can be found. The data which are made available are stored in two separate folders. The folder *low_performing* contains 1086 files, and the folder *well_performing* contains 1021 files. Each file is the simulation output for one building and resembles the whole-year energy and indoor climate measurements. To avoid confusion the files are named *lph* (low-performing house) and *wph* (well-performing house), and numbered creating a unique *houseID*. Three additional files contain the main building and household characteristics for each of the datasets and a common weather file. All files are provided in .csv format and the structure of the database is presented in Table 1.

**Table 1:** Structure of the dataset.

File name	Folder	Description
lph_parameters.csv	Districts	File including the main characteristics of the low-performing houses.
wph_parameters.csv	Districts	File including the main characteristics of the well-performing houses.
lph*n*.csv	low_performing	Simulation outputs for each low-performing house, where *n* is the identification number (houseID).
wph*n*.csv	well_performing	Simulation outputs for each well-performing house, where *n* is the identification number (houseID).
weather_data.csv	Districts	Weather file comprising outdoor boundary conditions used in simulations.

Table 2 shows the structure of files *lph_parameters.csv* and *wph_parameters.csv*. In these files, the properties for the houses and households can be found under the respective *houseID*-s. The meaning behind each listed property is briefly described in the table. It is important to note that the theoretical UA value for each building was calculated as the sum of the building elements’ area multiplied by the corresponding thermal transmittances. Since the ground floor is not in contact with the outdoors directly a weighting factor was calculated according to the standard ISO 13370:2017, while party walls are not included in this value.

**Table 2:** Description of the file including the main parameters for each household in the two generated districts.

Variable	Unit	Description
houseID		House identification number starts with *lph* for low-performing and with *wph* for well-performing houses.
construction_year		Allocated year of construction.
number_of_neighbours		Number of adjacent neighbours (0 for detached houses, 1 for semi-detached and >2 for terraced).
number_of_floors		Number of floors (in case of 1 the house is considered a single zone).
ground_floor_area	m^2^	Area of the ground floor (in case of single zone houses equals the total area, for others equals the day zone area).
other_floor_area	m^2^	Area of all other floors except the ground floor (equal to the area of the night zone).
outerwall_area	m^2^	Total area of the outer walls in contact with the outdoor environment.
window_area	m^2^	Total area of the windows.
roof_area	m^2^	Total area of the roofs.
partywall_area	m^2^	Total area shared with adjacent neighbours (assumed no heat transfer through this element).
total_volume	m^3^	Indoor air volume of the whole building.
heat_generator_type		Type of gas boiler (condensing or regular/non-condensing).
dhw_generator_type		Domestic hot water generation (“no” for houses in which DHW is not produced by the main boiler, “direct” for boilers with instantaneous production and “tank” for systems with storage tanks.
n50	h^−1^	n50 value.
household_size		Number of occupants.
total_presence	h	Number of hours the occupants were at home.
UA	W/K	Theoretical UA value is calculated based on the sampled U values and areas of heat loss, including an adjustment factor for the ground losses.

For the simulation a larger set of inputs was used for each house, however, given the source being a third party the authors could not disclose more information. Moreover, the authors consider the focus of this dataset to be the simulation outputs and not inputs, which are provided mainly informatively.

In Table 3, the outdoor environment variables which served as simulation input for both districts are listed. The weather conditions were measured for the location of Heverlee, Belgium during the year 2016. The measurements were collected as part of the research conducted at the Building Physics and Sustainable Design Section at KU Leuven.

**Table 3:** Description of the weather data file.

Variable	Unit	Description
Time	s	Simulation time logged in seconds in 10-minute timesteps.
Te	°C	Dry bulb temperature.
Te_dew	°C	Dew point temperature.
Humidity	%	Relative humidity.
Pressure	Pa	Atmospheric station pressure.
extra_hor	Wh/m^2^	Extraterrestrial horizontal radiation.
extra_dir_norm	Wh/m^2^	Extraterrestrial direct normal radiation.
infrared_intensity	Wh/m^2^	Horizontal infrared radiation intensity.
GHI	Wh/m^2^	Global horizontal radiation.
DNI	Wh/m^2^	Direct normal radiation.
diff_hor_rad	Wh/m^2^	Diffuse horizontal radiation.
win_dir		Wind direction (N=0, E=90, S=180, W=270).
win_speed	m/s	Wind speed.

Finally, Table 4 comprises the variables selected as outputs of the large-scale district simulations. Each of these files is named after the corresponding *houseID*. While analysing the data it is important to know that the number and name of variables differ for single-zone and two-zone buildings, the inputs which differ are indicated in the table.

**Table 4:** Description of the output variables for each simulated house.

Variable	Unit	Description
time	s	Simulation time logged in seconds in 10-minute timesteps.
T_day_K	K	Temperature measurement in the day zone.
T_night_K	K	Temperature measurement in the night zone.
T_k	K	Temperature measurement for houses modelled as a single zone.
q_day_W	W	Net energy demand of the day zone.
q_night_W	W	Net energy demand of the night zone.
q_W	W	Net energy demand for houses modelled as a single zone.
qDHW_W	W	Domestic hot water energy demand including distribution and storage efficiency.
qSH_W	W	Space heating energy demand including emission, distribution and control efficiency.
q_tot_W	W	Energy demand for space heating and domestic hot water including emission, distribution, storage and control efficiency.
q_gross_W	W	Energy demand for space heating and domestic hot water including all efficiency calculated with the load-dependent production efficiency.
inf_day_W	W	Infiltration losses in the day zone.
inf_night_W	W	Infiltration losses in the night zone.
inf_W	W	Infiltration losses for houses modelled as single zone.
gain_day_W	W	Internal gains in the day zone.
gain_night_W	W	Internal gains in the night zone.
gain_W	W	Internal gains for houses modelled as single zone.
sol_day_W	W	Solar gains in the day zone.
sol_night_W	W	Solar gains in the night zone.
sol_W	W	Solar gains for houses modelled as single zone.

The simulation outputs contain the information on the indoor temperature (*T_day_K, T_night_K, T_k*), net heating demand (*q_day_W, q_night_W, q_W*), infiltration losses (*inf_day_W, inf_night_W, inf_W*), internal gains (*int_day_W, int_night_W, int_W*) and solar gains for each zone (*sol_day_W, sol_night_W, sol_W*). On top of that additional post-processed data is provided when assuming the efficiency of different gas-fired system configurations. The total net heating demand for two-zone houses is calculated as the sum of the day and night zone individual demand or as the single-zone demand. This value is subsequently used as a base in postprocessing the space heating (SH) energy demand including emission, distribution and control efficiency (*qSH_W*). On the other hand, domestic hot water energy demand is retrieved from the occupancy profiles and includes distribution and storage efficiency (*qDHW_W*). When those two demands are summed they correspond to the total energy demand (*q_tot_W*), and after computing the load-dependent boiler production demand, the gross demand per house is obtained (*q_gross_W*).

## 4 Experimental Design, Materials and Methods

The principal tool which served for the generation of the dataset is the urban building energy modelling open tool TEASER [4] developed at RWTH Aachen. TEASER is a Python tool that transforms CityGML files into building energy simulation files compatible with the Modelica language, which contain structural, semantic and feature information. The underlying building geometries, derived from city scans, typically follow either Level of Detail (LOD) 1 (simplified cubic building shapes) or LOD 2 (enhanced with roof slopes). These models include essential parameters such as building geometry, function, construction year, number of floors, and height. TEASER processes the building geometry, assigning attributes like areas, orientations, and slopes to corresponding components (e.g., roof, wall, floor). For buildings constructed prior to 2006, before the EPBD [6], the dataset relied on the methodology presented in [5]. The modelled four key building components were: external walls, windows, roofs, and floors to the ground, and their properties were assigned according to construction periods. Data from EPCs for Flemish houses provided element-specific values, categorized into four periods: pre-1945, 1946-1970, 1971-1990, and 1991-2005. Although these buildings predated the latest European building directives, some included retrofitting initiatives documented in their EPCs. Figure 2 in [1] shows distributions of thermal transmittances (U-value) in [W/m^2^K] for the four building elements.

For constructions after 2006, building characteristics were derived from EPB reports in Flanders [6]. These reports provide annual average U-values and regulatory maximums for the four modelled building elements. To introduce variability, normal distributions were fitted around annual mean values, with standard deviations capped by legislative limits. This approach ensured that 99.7% of allocated values met the regulatory standards. Additionally, when transitioning between standard improvements, the standard deviation from the first year of a new regulation was adopted consistently. Table 1 in [1] summarizes the distributions for the four key building components. Unlike for buildings built prior to 2006, this dataset focused on smaller homes (under 200 m²) to better capture the influence of unmetered heat sources (e.g. internal or solar gains).

Based on the building component properties selected in the previous step, TEASER exports ready-to-run simulation models. In this case, models are outputted using the Integrated District Energy Assessment Simulations (IDEAS) library [7]. The IDEAS template used as the basis for this work consisted of the components for 1) building structure, 2) occupancy profiles, 3) infiltration model, and 4) heating system. The IDEAS version used in this work is IDEAS 2.1.0, dating June 4^th^, 2020 (https://github.com/open-ideas/IDEAS). An explanation of each of the four model's components follows.

First, the building structure comprised of four building elements (window, roof, external wall, and floor to the ground) is coupled to one or two thermal zones depending on the building geometry. Single-storey houses were modelled with a single zone, while houses with 2 or more storeys included distinct day and night zones, where the upper floors were modelled into one night zone. The day zone comprises spaces such as the kitchen, living room and bathroom, while the night zone consists of bedrooms. Detached, semi-detached, and terraced typologies were represented, and the existing party walls were treated as separate building elements. However, thermal exchange through party walls was excluded from the model, and they contributed to the building's thermal mass only. At the same time, internal wall areas were estimated to be equal to the sum of façade and party-wall areas. Heat exchange through the ground slab was incorporated using a uniform ground temperature assumption. Given the prevalence of heavyweight construction in Flanders, lightweight structures were excluded. Therefore, building envelopes shared a consistent material layering (Table 5), with thermal transmittance adjusted by modifying layer thicknesses. Window areas, not included in LOD 2 geometry, were estimated using window-to-wall ratios characteristic to building periods, and their thermal properties were based on predefined glazing and frame combinations. Here, several glazing and frame properties were adjusted to match the desired U-value together with a fixed frame-to-window ratio of 20%.

**Table 5:** Material layers used for the construction elements according to [5]. The materials are listed from inside to outside.

Building element	U-value [W/m^2^K]	Material type	Initial thickness [m]	Adapted in thickness
Roof	3.37	Gypsum plaster	0.01	
		Timber	0.01	
		Mineral wool	0.00	✓
External wall	2.52	Gypsum plaster	0.02	
		Heavy masonry	0.10	✓
		Mineral wool	0.00	✓
		Non-ventilated air cavity	0.00	✓
		Heavy masonry	0.09	
Ground floor	2.75	Ceramic tile	0.02	
		Screed	0.02	
		Expanded polystyrene	0.00	✓
		Dense cast concrete	0.14	
Internal wall	3.97	Gypsum plaster	0.02	
		Medium masonry	0.10	
		Gypsum plaster	0.02	
Party wall	3.42	Gypsum plaster	0.02	
		Medium masonry	0.14	

Second, occupant behaviour was modelled using the Event-based Residential Occupant Behaviour (EROB) framework [8], which takes into account occupancy patterns with employment types and daily schedules (i.e. full and part-time employed, unemployed, retired, student, and school-type occupants). For each of the occupants, three possible states were defined: present and awake, present and asleep, or away from home and the states were assigned according to the day of the week to reflect typical patterns. Activities were categorized and allocated based on their location and energy consumption, such as showering in the bathroom or cooking in the kitchen, and further divided into household tasks (e.g., doing laundry) and personal activities (e.g., using a computer). The profiles were developed using probabilities for starting states, activity durations, and transition likelihoods between activities. Based on these activities, the metabolic rate for each occupant was calculated, enabling the determination of internal heat gains. Additionally, internal heat contributions from appliances, such as fridges, freezers, televisions, laptops, smartphones, audio systems, irons, vacuum cleaners, dishwashers, stoves, ovens, microwaves, washing machines, and dryers were included, along with heat gains from lighting. A total of seven basic heating profiles were defined based on surveys conducted in the Netherlands. These profiles accounted for occupancy states and user preferences, such as which rooms were heated and their respective temperatures. A minimum temperature of 12°C was maintained in unheated zones. The heating demand for DHW was calculated similarly to appliance usage, with activities like showering, bathing, dishwashing, and others modelled based on their respective duration probabilities. Each zone's profile incorporated convective and radiative heat gains along with a temperature setpoint, while DHW demand was defined at the building level. For this study, 1,000 event-based occupant behaviour profiles were generated and randomly assigned to individual buildings.

To get insight into the main characteristics of the created occupancy profiles, consult Figure 4 in [1]. The profiles used in this work were created using the EROB version (https://github.com/siverbru/EROB) updated on September 30^th^, 2021.

Third, infiltration was modelled using the Alberta Air Infiltration Model (AIM-2) [9] and was feasible to compute without adding significant computational effort to the large-scale simulation. This model uses weather as the driving factor, with infiltration flow determined by superposing the temperature difference between indoors and outdoors and wind speed. Within the Modelica framework, the AIM-2 model's original equations were tailored to align with the building components in the IDEAS library. The two key parameters defining the building-specific infiltration rate were the pressure exponent *n* and the *n*_50_ pressurization test value. For houses built before 2006 the *n*_50_ was based on [10], *n*_50_ for houses built after 2006 on [6], and values for *n* were taken for all buildings from [11] since there is no evident relation found between *n* and building year. Mechanical ventilation was not included, ensuring the model's air exchange was done solely by natural infiltration. The distributions for parameters *n* and *n*_50_ are shown in Figure 3 in [1].

Fourth, this study assumed that the space heating (SH) and domestic hot water demand for buildings in the observed districts were post-processed, as is commonly done in UBEM. The methodology behind this postprocessing approach was developed by the authors, And the detailed mathematical annotation and an application example of this methodology can be found in [12]. As a base for the postprocessing, the net SH demand profile is outputted from the simulation, which assumes an ideal radiator heating system with 30% radiative and 70% convective heat transfer. During the simulation, the maximal available power of this idealized system corresponds to the designed power load of the house, calculated in accordance with NBN EN 12831 [13].

During postprocessing, once the SH simulation output and DHW demand profile from EROB are incorporated, a specific heating system is selected from six common types found in Flemish houses Two types of heat generators are considered: non-condensing and condensing gas boilers, each matched to one of three DHW production subsystems. These subsystems include systems where DHW is independent of the primary heat generator, systems with instantaneous DHW production (combi boilers), and indirect systems utilizing DHW buffer tanks. In the subsequent step, emission and distribution efficiencies are incorporated into the SH demand. Following this, DHW demand is computed, accounting also for distribution and storage efficiencies. In the end, the SH and DHW profiles are combined, and a probabilistically sampled load-dependent production efficiency is added to determine the boiler's total energy consumption. The designed power of the heating system is assigned: 32 kW or 45 kW for low-performing houses and 15 kW for high-performing houses, regardless of building characteristics as it is commonly done in practice. For buildings constructed before 2006, all six system variations are applied, while for those built after 2006, only the three variants associated with condensing gas boilers are utilized. The distribution of these systems is illustrated in Figure 5 in [1]. The exported Modelica building models were simulated over a full calendar year with a time step of 10 minutes, preceded by a two-week initialization period. These simulations were carried out for the year 2016, at the location of Heverlee, Belgium. Simulations are executed in Dymola 2017 along with the Modelica Standard Library 3.2.2. The version of TEASER utilized as the base was TEASER version 0.5.2, dated July 13^th^, 2017.

After the described simulation and postprocessing, the data was arranged using R Studio software in individual .csv files for each house, and an overview document.

## Limitations

The building stocks were developed as representative of Belgium and, despite the range of configurations, they comprise only the heavy-weight typology. Limitations concerning the buildings’ envelope are that the heat transfer through party walls to adjacent buildings was not simulated, and heat losses to the ground were assumed to occur at a uniform temperature. Another aspect that is relevant in the representativeness of the building stocks compared to reality is the pairing of house type and size with the occupant behaviour, mainly operational set-points. Namely, when allocating the occupancy the selection was done randomly, therefore for some houses there is a possibility that extremely large houses, especially with large night zones, are paired with high heating set-points, which might not be the case in real life. Moreover, only gas heating systems were allocated in both building stocks while the inclusion of different heat sources (e.g. heat pumps) could provide a more realistic scenario when coupled with well-performing houses. While enabling lower computational effort, the postprocessing of the heating system enables the original dataset to be used for different purposes. Finally, the simulated datasets replicate ideal monitoring campaigns, with no measurement errors or data noise.

## Ethics Statement

The authors confirm that they have read and adhere to the ethical requirements for publication in Data in Brief. This work does not involve any human subjects, animal experiments, or data collected from social media platforms.

## CRediT Author Statement

**Katia Ritosa:** Conceptualization, Investigation, Methodology, Software, Validation, Data Curation, Formal Analysis, Writing - Original Draft. **Ina De Jaeger:** Conceptualization, Methodology, Software, Data Curation. **Dirk Saelens:** Conceptualization, Supervision, Funding acquisition, Writing- Reviewing and Editing. **Staf Roels:** Conceptualization, Supervision, Funding acquisition, Writing- Reviewing and Editing.

## Data Availability

ZenodoSimulated heating energy demand for two residential neighbourhoods (Original data).
